# CircDiaph3 aggravates H/R-induced cardiomyocyte apoptosis and inflammation through miR-338-3p/SRSF1 axis

**DOI:** 10.1007/s10863-023-09992-5

**Published:** 2024-04-13

**Authors:** Lin Lin, Li Wang, Aimin Li, Yanzhuo Li, Xiaolong Gu

**Affiliations:** 1 Department of Cardiovascular Medicine, PLA Southern Theater Command General Hospital, 11 Liuhua Road, Guangzhou, 510000 China; 2 Department of Emergency, PLA Southern Theater Command General Hospital, 11 Liuhua Road, Guangzhou, 510000 China

**Keywords:** Myocardial Infarction, CircDiaph3, miR-338-3p, Cardiomyocytes, Inflammation

## Abstract

Acute myocardial infarction (AMI) is one of the most prevalent cardiovascular diseases, accounting for a high incidence rate and high mortality worldwide. Hypoxia/reoxygenation (H/R)-induced myocardial cell injury is the main cause of AMI. Several studies have shown that circular RNA contributes significantly to the pathogenesis of AMI. Here, we established an AMI mouse model to investigate the effect of circDiaph3 in cardiac function and explore the functional role of circDiaph3 in H/R-induced cardiomyocyte injury and its molecular mechanism. Bioinformatics tool and RT-qPCR techniques were applied to detect circDiaph3 expression in human patient samples, heart tissues of AMI mice, and H/R-induced H9C2 cells. CCK-8 was used to examine cell viability, while annexin-V/PI staining was used to assess cell apoptosis. Myocardial reactive oxygen species (ROS) levels were detected by immunofluorescence. Western blot was used to detect the protein expression of anti-apoptotic Bcl-2 while pro-apoptotic Bax and cleaved-Caspase-3. Furthermore, ELISA was used to detect inflammatory cytokines production. While bioinformatics tool and RNA pull-down assay were used to verify the interaction between circDiaph3 and miR-338-3p. We found that circDiaph3 expression was high in AMI patients and mice, as well as in H/R-treated H9C2 cells. CircDiaph3 silencing ameliorated apoptosis and inflammatory response of cardiomyocytes in vivo. Moreover, the knockdown of cirDiaph3 mitigated H/R-induced apoptosis and the release of inflammatory mediators like IL-1β, IL-6, and TNF-α in H9C2 cells. Mechanistically, circDiaph3 induced cell apoptosis and inflammatory responses in H/R-treated H9C2 cells by sponging miR-338-3p. Overexpressing miR-338-3p in H/R-treated cells prominently reversed circDiaph3-induced effects. Notably, miR-338-3p inhibited SRSF1 expression in H/R-treated H9C2 cells. While overexpressing SRSF1 abrogated miR-338-3p-mediated alleviation of apoptosis and inflammation after H/R treatment. To summarize, circDiaph3 aggravates H/R-induced cardiomyocyte apoptosis and inflammation through the miR-338-3p/SRSF1 axis. These findings suggest that the circDiaph3/miR-338-3pp/SRSF1 axis could be a potential therapeutic target for treating H/R-induced myocardial injury.

## Introduction

Acute myocardial infarction (AMI) is one of the most common cardiovascular diseases, accounting for millions of deaths globally (Tsao et al. 2022). In this context, the myocardial ischemia-reperfusion injury is an important pathological process of myocardial injury after AMI (Neri et al. 2017). Myocardial ischemia and ventricular remodeling can substantially induce myocardial cell injury (Jennings 2013). Moreover, the oxidative phosphorylation of mitochondria in cardiomyocytes is halted during hypoxia, leading to myocardial injury (Ham and Raju 2017). However, the mechanism of myocardial injury has not been fully elucidated at a molecular level. Indeed, inflammation plays an important role in the pathogenesis of H/R injury, leading to cardiomyocyte apoptosis (Castillo et al. 2020). Therefore, the effective reduction in the levels of apoptosis and inflammatory responses is of great significance in reducing myocardial injury after myocardial infarction.

Circular RNAs (circRNAs) play important regulatory roles in many cardiovascular diseases including AMI, which may become specific markers for clinical detection (Tang et al. 2021; Zhao et al. 2020). Previous studies demonstrated that most of the circRNAs could effectively regulate the expression of downstream target genes through the competitive binding of miRNAs, thereby affecting the biological function of the body (Zhao et al. 2020; Li et al. 2021, 2023). Notably, it was observed that the expression of heart-related circRNA (HRCR) is downregulated during heart failure in mice (Li et al. 2023). HRCR can competitively adsorb miRNA-223 and inhibit its expression, thereby promoting the expression of downstream target genes and inhibiting the progress of heart failure (Wang et al. 2016). Several reports indicated that circRNAs are involved in the regulation of myocardial injury by sponging various miRNAs (Yin et al. 2022; Zhang et al. 2022a, b). Nonetheless, the role of circDiaph3 in myocardial injury remains unclear. At present, many circRNAs function in cardiomyocytes by interacting with related miRNAs. However, the role of circDiaph3 in H/R-induced cardiomyocytes is still unclear. Therefore, in the present report, we investigated the role of circDiaph3 and its molecular mechanism in regulating H/R-induced cardiomyocyte apoptosis and inflammation.

## Materials and methods

### Bioinformatic analysis

The Gene Expression Omnibus (GEO) Database with accession number GSE160717 was used to download the circRNA expression data for three AMI patients and three healthy controls (Zhao et al. 2021). The differentially expressed circRNAs between AMI patients and controls and the clinical information of patients and normal controls are summarized in the previous publication (Zhao et al. 2021).

### Myocardial I/R mouse model

The Model Animal Research Institute of PLA Southern Theater Command General Hospital provided 20 male C57BL/6J mice used in this study. The mice were randomly divided into two groups: sham group (n = 10) and AMI group (n = 10). The AMI mouse model was developed as previously mentioned (Wang et al. 2021). Briefly, Pentobarbital sodium (50 mg/kg, P3761, Sigma-Aldrich) was administered intraperitoneally to anaesthetize mice and then cut the left sternum to fully expose the heart. To induce ischemia, a 5 to 0 (2 mm) suture was used to ligate the left anterior descending (LAD) coronary artery. Electrocardiographic ST elevation was used to confirm AMI. The sham group consisted of mice with the same surgical procedure except without ligation of the anterior descending branch of the left coronary artery. ShRNA-carrying adenovirus against circDiaph3 was myocardially injected into mice to construct a model of AMI + si-circDiaph3, while the comparable control was AMI + si-NC. After two days of surgery, mice were euthanized by increasing the CO_2_ flow into their cages to a rate of 3 L/min until they die. Subsequently, hearts were collected for assays.

### Cell culture

Rat cardiomyocyte H9C2 cells were purchased from the Type Culture Collection of the Chinese Academy of Sciences (Shanghai, China). The cells were maintained in DMEM medium (Gibco Healthcare Life Sciences, Logan, UT, USA) with 10% fetal bovine serum (FBS, Thermo Fisher Scientific, Inc., Waltham, MA, USA). In the control group, H9C2 cells were cultured at 37 °C and 5% CO_2_. To construct a cell model of myocardial H/R injury, H9C2 cells were kept under a hypoxic condition (95% N2 and 5% CO2 at 37 °C) for 2 h and then reoxygenated (75% N2, 20% O2 and 5% CO2) for 12 h.

### Cell transfection

H9C2 cells were initially seeded at a density of 1 × 10^5^ cells/well in 6-well plates and incubated at 37°C overnight until about 60% density. After replenishing with the serum-free medium, the cells were transfected with si-NC (negative control) sense, 5’-UUCUCCGAACGUGUCACGUTT-3’ and antisense, 5’-ACGUGACACGUUCG GAGAATT-3’ or si-circDiaph3 sense, 5’-CGGCAGGCAUUAGAGAUGAACAGCA − 3’ and antisense, 5’-UGCUGUUCAUCUCUAAUGCCUGCCG-3’ using the Lipofectamine® 2000 (Invitrogen; Thermo Fisher Scientific, Inc.), or with miR-338-3p inhibitor (Cat; 4,464,079, Life Technologies) using Lipofectamine RNAiMAX Transfection Reagent (Invitrogen), according to the manufacturer’s protocol. Cells that did not need H/R treatment were transfected for 48 h, while cells that did require H/R treatment were treated for 48 h after transfection. The cells were then harvested for subsequent assays.

### Cell proliferation assay

The viability of H9C2 cells was measured by Cell Counting Kit (CCK)-8 Assay Kit (Solarbio, M1020-500T). Briefly, the cells were seeded in the 96-well plates at a density of 5 × 10^3^ cells/well and incubated at 37 °C for predetermined time intervals of 24, 48, and 72 h. After incubation, 10 µl of CCK-8 working reagent was added to each well and incubated for 30 min. Finally, a final concentration of 10% Sodium dodecyl sulfate (SDS, Solarbio, S8010) was added to each well, and the optical density (OD) values at 570 nm were recorded.

### Apoptosis assay

We used the Annexin V-FITC apoptosis detection kit (Sigma-Aldrich, St. Louis, MO) to study apoptosis. Shortly, cardiac cells and H9C2 cells were collected and resuspended in 1 suspended in 1× binding buffer and stained with Annexin V-FITC (Elabscience, China) and propidium iodide (Elabscience, China) for 20 min at room temperature under dark conditions. Finally, the cells were acquired by FACSCanto II flow cytometry (BD Biosciences) and analyzed with FlowJo software.

### Immunofluorescence detection of myocardial ROS levels

For evaluating the oxidative stress level in H9C2 cells, the DCFDA/H2DCFDA - Cellular ROS Assay Kit (ab113581, Abcam) was used. Briefly, H9C2 cells were plated into a 6-well plate at a density of 3 × 10^5^/well. After indicated treatment, cells were washed with PBS and then stained with 2′,7′-dichlorofluorescin diacetate (DCFDA) solution (20 µM) for 45 min at 37 °C. The nuclei were stained with DAPI solution. The fluorescence signal was detected and observed using fluorescence microscope (BX53, Japan) and images were acquired. The intracellular ROS levels were observed in red color, while the nuclei of the cells appeared blue under the microscope. The Indica Labs - Area Quantification FL v2.1.2 module in Halo v3.0.311.314 analysis software was used to quantify the target area of each section separately, and the mean intensity was calculated.

### Western blotting

Proteins from cardiomyocytes and H9C2 cells were extracted with RIPA Lysis buffer (Beyotime Biotechnology) containing Protease Inhibitor Cocktail (Roche Applied Science, Pleasanton, CA, USA). The total protein concentration was measured by BCA Protein Assay Kit (Solarbio, PC0020) and then loaded onto sodium dodecyl sulfate-10% polyacrylamide gel for electrophoresis (SDS-PAGE). The proteins were then transferred to Immobilon-NC Membranes (Triton-free, mixed cellulose esters, 0.45 μm, Solarbio, YA1711). Thereafter, the membranes were incubated with primary antibodies against BAX, BCL-2, cleaved-caspase-3, SRSF1, and GAPDH (Abcam) overnight at 4℃. Next day, the membranes were incubated with HRP-labeled secondary antibody and finally, the expression of the relative protein was detected with ECL chemiluminescent solution. The expression level of specific protein was normalized to GAPDH level and quantified using Image LabTM Software (Bio-Rad, Shanghai, China).

### RNA isolation, cDNA synthesis, and real-time- qPCR

Total RNA from cardiomyocytes and H9C2 cells was extracted with Trizol™ Plus RNA Kit (ThermoFisher Scientific, IS10007, Waltham USA) and then reverse transcribed into cDNA (Superscript II reverse transcriptase, Life Technologies). The relative levels of circDiaph3, miR-338-3p, IL-1β, IL-6, TNF-α, and β-actin were determined with FastFire qPCR PreMix (SYBR Green, Tiangen, FP207, China) in a ProFlex™ PCR system (ThermoFisher Scientific) using the standard curve method. Each PCR mixture was initially denatured at 95 °C for 5 min and then cycled 40 times at 95 °C for 10 s, 60 °C for 15 s, and 72 °C for 8 s. The expression of genes was normalized to β-actin or U6 and calculated by 2^−ΔΔCt^ method. Primer sequences used in this study are given in Table 1.

**Table 1:** Primer sequences used in the study

Primer name	Sequence (5’-3’)
circDiaph3 (Forward)	TGAATAACTTCAGAACCACATT
circDiaph3 (Reverse)	CTCCTGTCTCATCACCCT
miR-338-3p (Forward)	ATCCAGTGCGTCTCGTG
miR-338-3p (Reverse)	GTCGTTGCTTGGTTCTCCTTGT
IL-1β (Forward)	GCACTACAGGCTCCGAGATGAA
IL-1β (Reverse)	GTCGTTGCTTGGTTCTCCTTGT
IL-6 (Forward)	CTTGGGACTGATGCTGGTGACA
IL-6 (Reverse) TNF-α (Forward) TNF-α (Reverse)	GCCTCCGACTTGTGAAGTGGTA CCGCTCGTTGCCAATAGTGATG CATGCCGTTGGCCAGGAGGG
β-actin (Forward)	AGCCACATCGCTCAGACAC
β-actin (Reverse)	GCCCAATACGACCAAATCC
U6 (Forward)	TGCTATCACTTCAGCAGCA
U6 (Reverse)	GAGGTCATGCTAATCTTCTCTG

### ELISA

The supernatant of the cell culture medium was collected and then centrifuged at 2458 g, for 15 min. Commercial kits were used to measure the levels of IL-1β, IL-6, and TNF-α according to the manufacturer’s instructions (R&D Systems).

### Dual luciferase activity assay

The StarBase online tool was used to predict the binding site of the circDiaph3 and miR-338-3p or SRSF1 and miR-338-3p. Luciferase reporters were constructed via cloning wildtype (WT) or mutant (MUT) potential binding sites for miR-338-3p in circDiaph3 or the 3’UTR of SRSF1 into the pmirGLO vectors (Promega). H9C2 cells were then co-transfected with either negative control (NC) mimic (miR-NC) or with miR-338-3p mimics and WT or MUT luciferase reporters (circDiaph3-WT, circDiaph3-MUT, SRSF1-3’UTR-WT, and SRSF1-3’UTR-MUT) for 48 h. Thereafter, the cells were harvested, and the luciferase activity was detected using the Dual-Luciferase Reporter Assay Kit (Promega).

### RNA pull-down

RNA pulldown assay was performed with biotinylated antisense oligos. In brief, H9C2 cells were rinsed with cold PBS once, and resuspended in RIPA buffer for 10 min. Thereafter, cells were harvested and sonicated for 10 min, and then centrifugated at 13,000 rpm for 20 min. Then the supernatant was added with 100 pmol probes and cultured for 2 h at 4 °C. The magnetic RNA-protein pulldown kit (Thermo, Waltham, MA, USA) was used to pull down the biotin-coupled RNA complexes in accordance with the manufacturer’s instructions. 3′ end biotin-labeled circDiaph3 (Bio-circ_ circDiaph3) together with Bio-NCs (negative controls mixed with streptavidin magnetic beads) were then co-incubated with cell lysates at 4 °C overnight. After washing with buffer, RT-qPCR was carried out to measure the abundance of coprecipitated RNAs.

### Statistical analysis

Data were presented as mean ± standard deviation (S.D.), and the analyses were conducted with SPSS 22.0 software (SPSS Inc., Chicago, US). Notably, the differences between groups were compared with the student’s two-tailed *t*-test and analysis of variance (ANOVA) followed by the post-hoc test at a defined level of statistical significance of *P* < 0.05.

## Results

### Increased level of circDiaph3 in cardiomyocytes of AMI mouse model

**Fig. 1:**
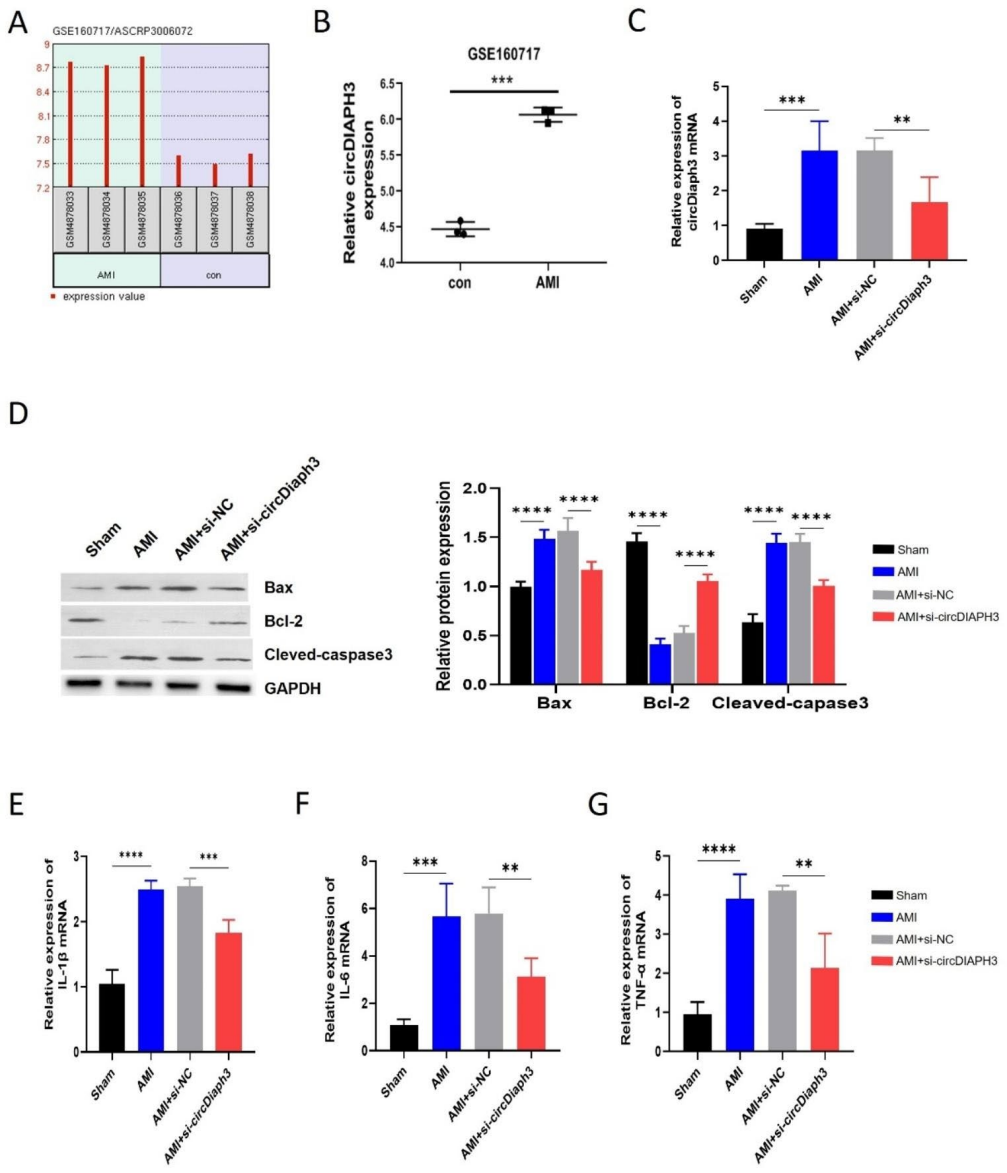
Expression of circDiaph3 in AMI patients and mouse model. **(A-B)** GSE160717 database was used to analysis circDiaph3 expression in AMI patients and normal healthy controls (AMI patients = 3, normal controls = 3). **(C)** RT-qPCR was used to detect the expression of circDiaph3 in cardiomyocytes of AMI mouse model in indicated groups (n = 10). **(D)** Western blot was used to detect the protein expression levels of Bax, Bcl-2, and cleaved-Caspase-3 in cardiomyocytes in indicated groups. RT-qPCR was used to detect the gene expression levels of (E) IL-1β; **(F)** IL-6; **(G)** TNF-α in cardiomyocytes in indicated groups. **P < 0.01; ***P < 0.001 and ****P < 0.0001

To investigate the role of circDiaph3 in AMI, we first evaluated the expression of circDiaph3 in healthy control and AMI patients using bioinformatics online tool (GEO accession: GSE160717, https://www.ncbi.nlm.nih.gov/geo/query/acc.cgi?acc=GSE160717). Results showed that circDiaph3 was highly expressed in AMI patients compared to the healthy control (Fig. 1A-B). Then, using a mouse model of AMI, we discovered that mice with AMI had higher circDiaph3 expression level in contrast to the sham control group mice (Fig. 1C). Moreover, there was no discernible difference in circDiaph3 expression levels between the AMI and si-NC groups. However, when AMI mice were injected with shRNA-carrying adenovirus against circDiaph3, the expression of circDiaph3 was drastically reduced compared to the control si-NC group (Fig. 1C). These findings imply that circDiaph3 expression is high and may play an important role in AMI.

### circDiaph3 promotes apoptosis and inflammation of cardiomyocytes in vivo

In order to explore the function of circDiaph3 in AMI, we observed the apoptosis of cells by measuring apoptosis-related proteins i.e., Bcl-2, Bax, and cleaved-Caspase-3 using western blot. Compared with the sham group, the expression of anti-apoptotic protein Bcl-2 expression was low while that of pro-apoptotic proteins Bax and cleaved caspase3 expression was high both in AMI and AMI + si-NC groups (Fig. 1D). Notably, knockdown of circDiaph3 prominently reduced the expression of Bax and cleaved caspase3 while promoted Bcl-2 expression (Fig. 1D).

We also investigated the role of circDiaph3 in AMI inflammation. Results demonstrated that the gene expression levels of inflammatory cytokines such as IL-1β, IL-6, and TNF- α were significantly high in AMI and AMI + si-NC groups compared to the sham group (Fig. 1E-G). Interestingly, the knockdown of circDiaph3 dramatically reduced the levels of these inflammatory cytokines. These data indicate that circDiaph3 could promote the apoptosis and inflammatory response of cardiomyocytes in AMI which can be alleviated by targeting circDiaph3.

### Knockdown of circDiaph3 inhibits H/R-induced apoptosis and inflammation in vitro

**Fig. 2:**
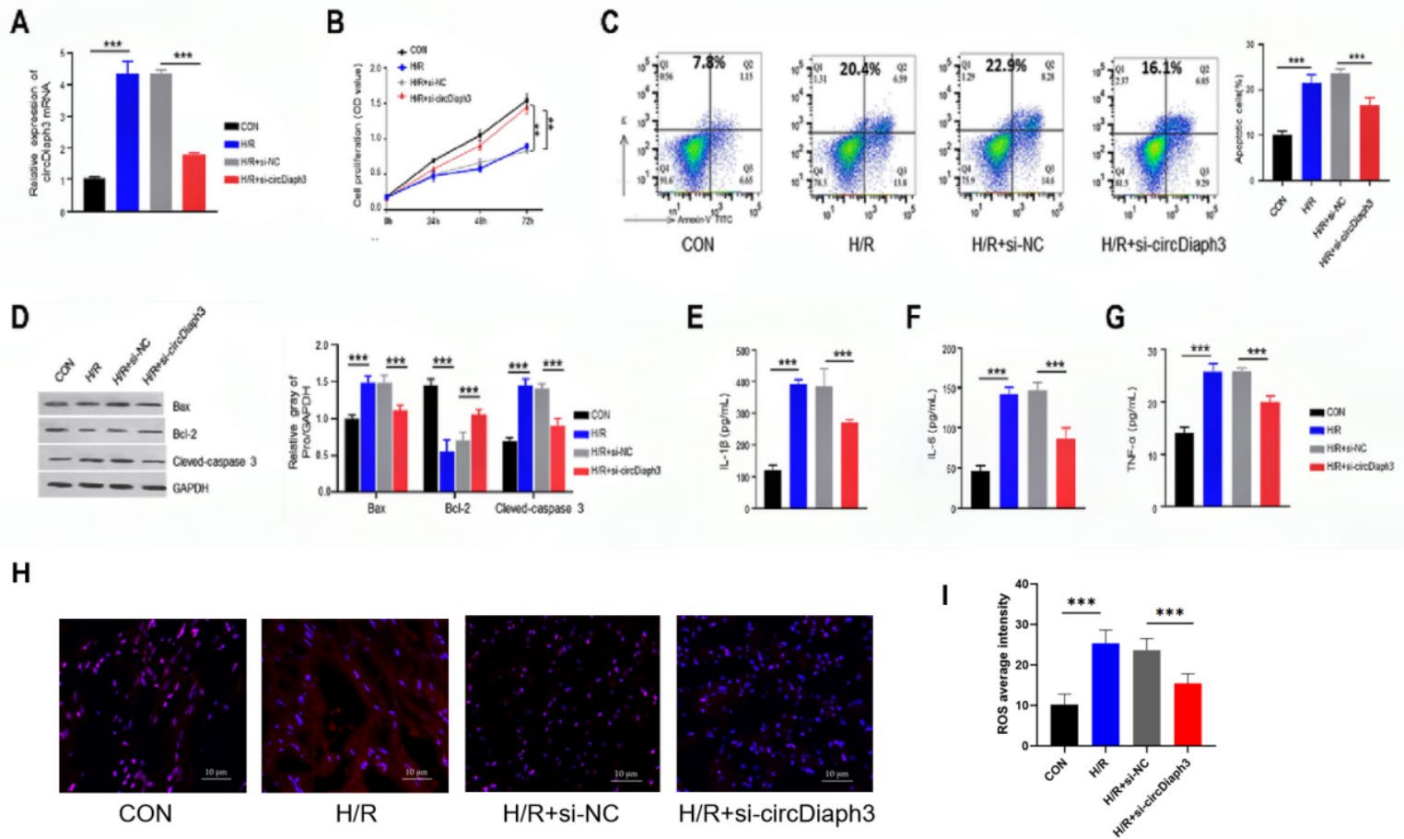
Silencing circDiaph3 inhibited H9C2 cell apoptosis and inflammatory reaction induced by H/R. **(A)** RT-qPCR was used to detect the expression of circDiaph3 in the indicated groups of H9C2 cells. **(B)** CCK-8 assay was used to detect the proliferation rate of H9C2 cells in different groups. **(C)** Annexin V-PI staining was used to detect apoptosis in different groups of H9C2 cells. **(D)** Western blot was used to detect the protein expression levels of Bax, Bcl-2, and cleaved-Caspase-3. Elisa was used to measure the concentration levels of **(E)** IL-1β; **(F)** IL-6; **(G)** TNF-α in different groups of H9C2 cells. **(H)** The ROS level in H9C2 cells was evaluated using immunofluorescence (× 400). **(I)** Relative intensity of ROS in each group. **P < 0.01; and ***P < 0.001

In the next step, we evaluated the expression of circDiaph3 in H/R-treated H9C2 cells, a cell model of myocardial H/R injury. RT-qPCR analysis revealed that compared with the control (CON) treatment group, the expression level of circDiaph3 in H9C2 cells induced by H/R was prominently high which was significantly reduced after knocking down circDiaph3 in H9C2 cells (Fig. 2A). We then evaluated the effects of inhibiting circDiaph3 on H/R-induced apoptosis and inflammation. For this purpose, circDiaph3 was knockdown in H9C2 cells. RT-qPCR was used to detect the expression level of circDiaph3 in different groups of H9C2 cells (Con, H/R, H/R + si-NC, and H/R + si-circDiaph3). After H/R induction, the expression level of circDiaph3 was significantly increased in H/R group, however, its level drastically decreased in H/R + si-circDiaph3 group after knocking down circDiaph3 (Fig. 2A). We then evaluated the effect of knocking down circDiaph3 on H9C2 cells viability in different treatment groups (con, H/R, H/R + si-NC, and H/R + si-circDiaph3) at different time points such as 0, 24, 48, and 72 h. H/R treatment significantly reduced H9C2 cells proliferation capability, while the transfected si-circDiaph3 cells exhibited high proliferation capability (Fig. 2B). Flow cytometry was used to detect the apoptosis level of H9C2 cells in different groups (con, H/R, H/R + si-NC, and H/R + si-circDiaph3) and we found increased apoptosis level in H/R group (22.31 ± 1.94), while the apoptosis level of si-circDiaph3 cells was decreased (16.25 ± 1.48) (Fig. 2C). Moreover, the protein expression levels of Bax, and cleaved-Caspase-3 were elevated while Bcl-2 protein level was downregulated in H/R group which were partially reversed by silencing circDiaph3 as shown in H/R + si-circDiaph3 group (Fig. 2D). Additionally, it was observed that the production levels of IL-1β, IL-6, and TNF- α were increased (380.23 ± 7.36, 144.26 ± 9.68, 26.34 ± 5.27, respectively) in H9C2 cells after H/R induction compared to the controls). However, silencing circDiaph3 significantly reduced these inflammatory mediators’ production in H9C2 cells (Fig. 2E-G). Moreover, we also evaluated the effect of circDiaph3 inhibition on ROS levels. After H/R induction, the expression level of the average intensity of ROS was significantly increased in the H/R group(25.36 ± 3.24), however, after knockdown of circDiaph3, its level decreased dramatically in the H/R + si-circDiaph3 group (15.47 ± 2.34) (Fig. 2H-I). Taken together, these findings suggest that circDiaph3 silencing reduced the inflammatory response and apoptosis in H/R-induced cardiomyocyte.

### circDiaph3 targets miR-338-3p

**Fig. 3:**
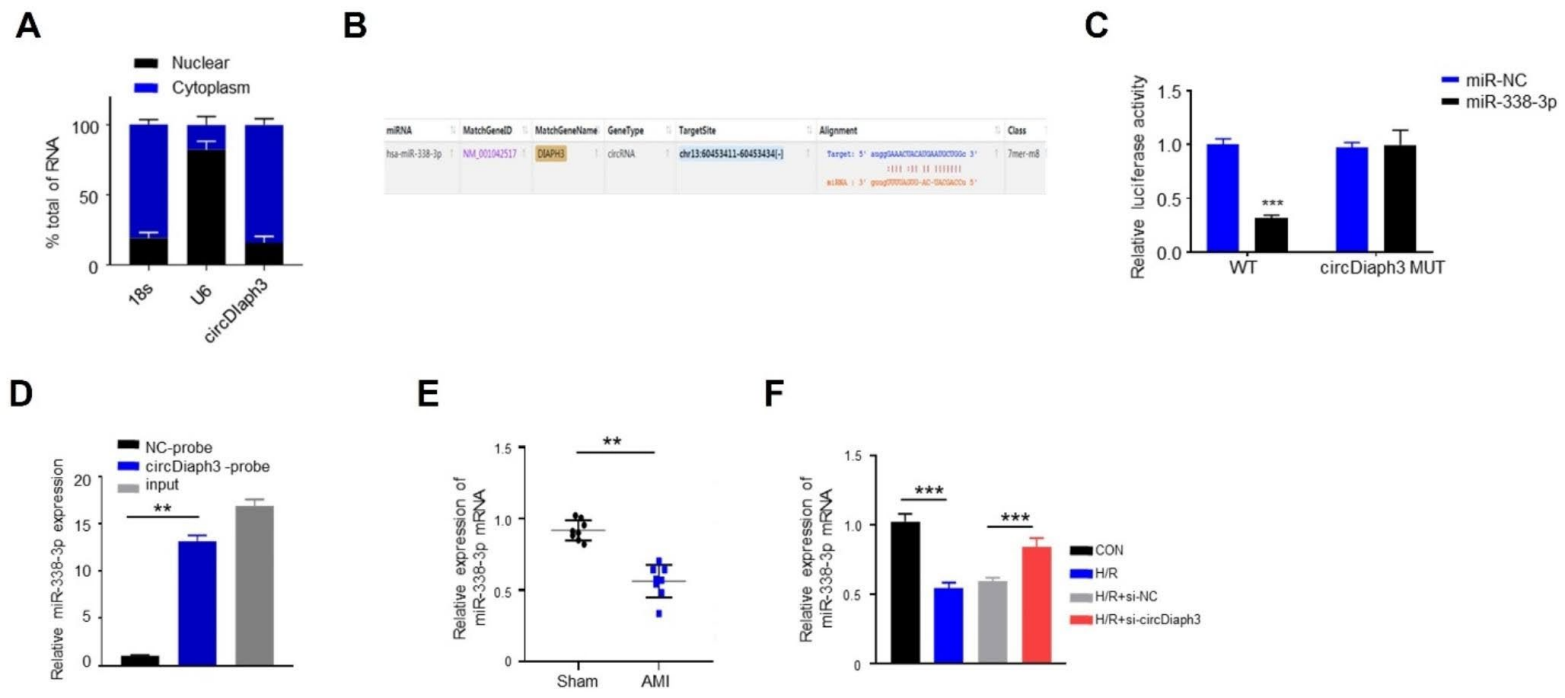
circDiaph3 targeted miR-338-3p. **(A)** The subcellular localization of circDiaph3 in H9C2 cells was detected by the nuclear-cytoplasmic separation. **(B)** Predicted and mutated binding sites for miR-338-3p in circDiaph3. **(C)** A dual-luciferase reporter technique was used to measure the relative luciferase activity of WT-circDiaph3 and MUT-circDiaph3 following transfection of miR-338-3p mimic or NC. **(D)** RNA pull-down assay was used to investigate the biding between circDiaph3 and miR-338-3p. **(E)** RT-qPCR was used to detect the expression of miR-338-3p in myocardial tissue of AMI mice and Sham mice. **(F)** RT-qPCR was used to detect the expression level of miR-338-3p in different groups of H9C2 cells. **P < 0.01; and ***P < 0.001

To investigate the mechanism by which circDiaph3 regulates cardiomyocyte apoptosis and inflammation, the subcellular localization of circDiaph3 in H9C2 cells was detected by the nuclear-cytoplasmic separation approach. The expression of circDiaph3 in the nucleus and cytoplasm was detected by RT-qPCR, in which circDiaph3 was mainly located in the cytoplasm, with U6 and 18 S being the internal reference controls for nucleus and cytoplasm, respectively (Fig. 3A). We then utilized bioinformatic StarBase tool to predict the binding target of circDiaph3. It was found that circDiaph3 targeted miR-338-3p (Fig. 3B). To verify this hypothesis, a luciferase reporter gene experiment was carried out in H9C2 cells and when compared to the miR-NC group, it was discovered that the relative luciferase activity of the miR-338-3p mimic was significantly reduced in the WT-circDiaph3 group (0.38 ± 0.05), but not in the MUT-circDiaph3 group (Fig. 3C). RNA pull-down assay in H9C2 cells further confirmed that circDiaph3 and miR-338-3p were directly bound, and circDiaph3 probe enriched more miR-338-3p than NC probe, as shown in Fig. 3D. Additionally, we examined the expression of miR-338-3p in AMI and sham mice and found that AMI mice had higher miR-338-3p expression compared to the sham control group mice (0.52 ± 0.6) (Fig. 3E). We further verified the expression level of miR-338-3p in different groups of H9C2 cells (con, H/R, H/R + si-NC, and H/R + si-circDiaph3). We noticed that miR-338-3p was dramatically downregulated in H/R-treated H9C2 cells, however, when circDiaph3 expression was silenced, the significant downregulation of miR-338-3p in H/R-treated H9C2 cells was partially restored (Fig. 3F). These findings demonstrate that miR-338-5p is the novel downstream target of circDiaph3.

### circDiaph3 suppresses H/R-induced H9C2 cell injury by sponging miR-338-3p

**Fig. 4:**
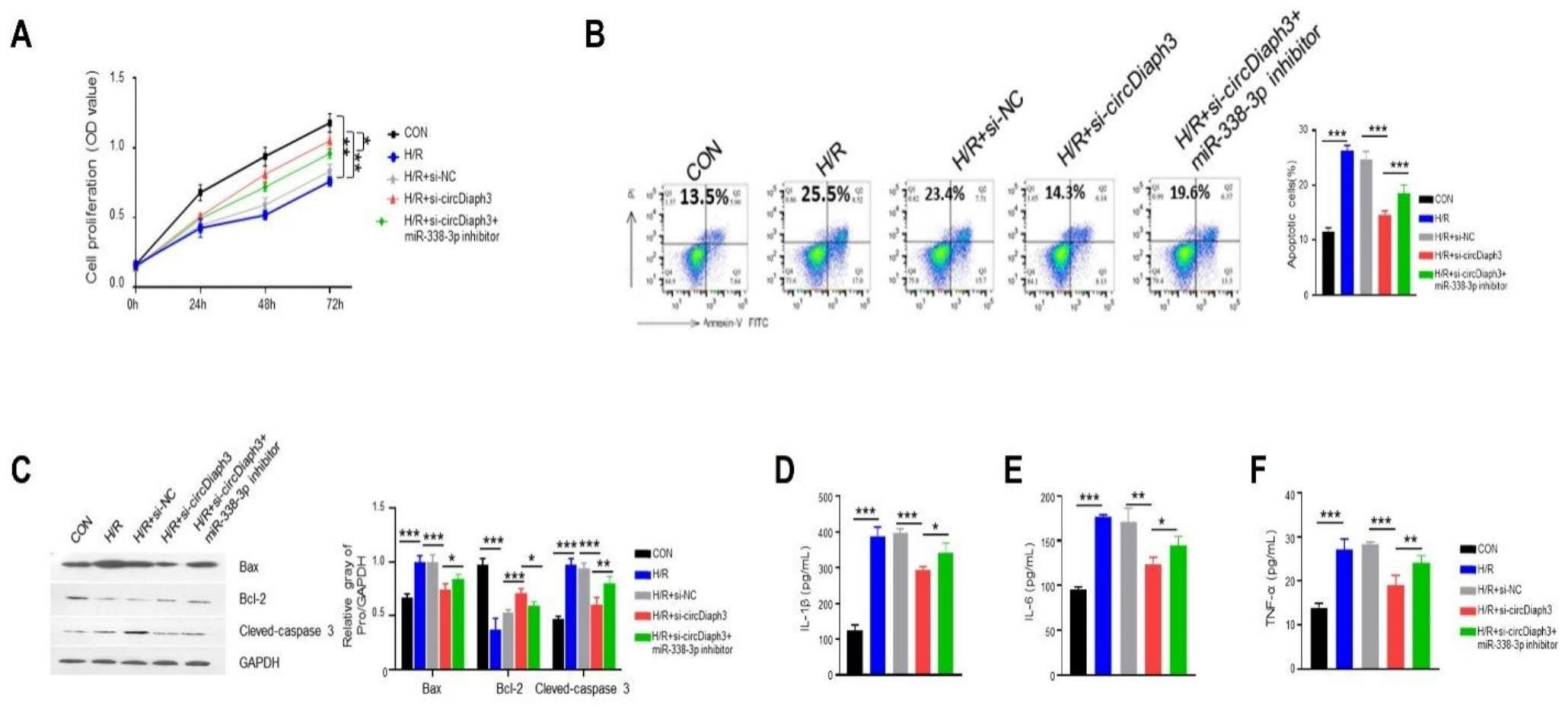
CircDiaph3 inhibited apoptosis and inflammation in H/R-treated cells by suppressing miR-338-3p expression. **(A)** CCK-8 assay was used to detect the proliferation rate of H9C2 cells in different groups. **(B)** Annexin V-PI staining was used to detect apoptosis in different groups of H9C2 cells. **(C)** Western blot was used to detect the protein expression levels of Bax, Bcl-2, and cleaved-Caspase-3. Elisa was used to measure the concentration levels of **(D)** IL-1β; **(E)** IL-6; **(F)** TNF-α in different groups of H9C2 cells. *P < 0.05; **P < 0.01; and ***P < 0.001

H9C2 cells were transfected with si-circDiaph3 alone or in combination with miR-338-3p inhibitor to examine whether circDiaph3-mediated suppression of H9C2 cell apoptosis and inflammatory response was dependent on miR-338-3p inhibition. Increased H9C2 cell proliferation caused by H/R treatment was reduced by silencing circDiaph3, but it was abrogated by concomitant inhibition of miR-338-3p (Fig. 4A). Flow cytometry was used to detect the apoptotic level of H9C2 cells in different groups. The apoptotic level of H9C2 cells (27.26 ± 4.83) was increased after H/R induction, which was reduced in si-circDiaph3 cells. Interestingly, H9C2 cells transfected with miR-338-3p inhibitor partially increased the apoptosis level (Fig. 4B). Additionally, in cells treated with H/R, silencing of circDiaph3 decreased the expression of cleaved-caspase 3 and Bax (0.76 ± 0.10, 0.80 ± 0.09, respectively) while increased the expression of Bcl-2 (1.50 ± 0.13); however, concurrent silencing of miR-338-3p reversed these effects (Fig. 4C). Furthermore, circDiaph3 silencing prevented H/R-induced IL-1β, IL-6, and TNF-α secretion, which was abolished by concomitant inhibition of miR-338-3p (Fig. 4D-F). These findings suggest that circDiaph3 inhibits H/R-induced apoptosis and inflammation by reducing the expression of miR-338-3p.

### Mir-338-3p targets SRSF1

**Fig. 5:**
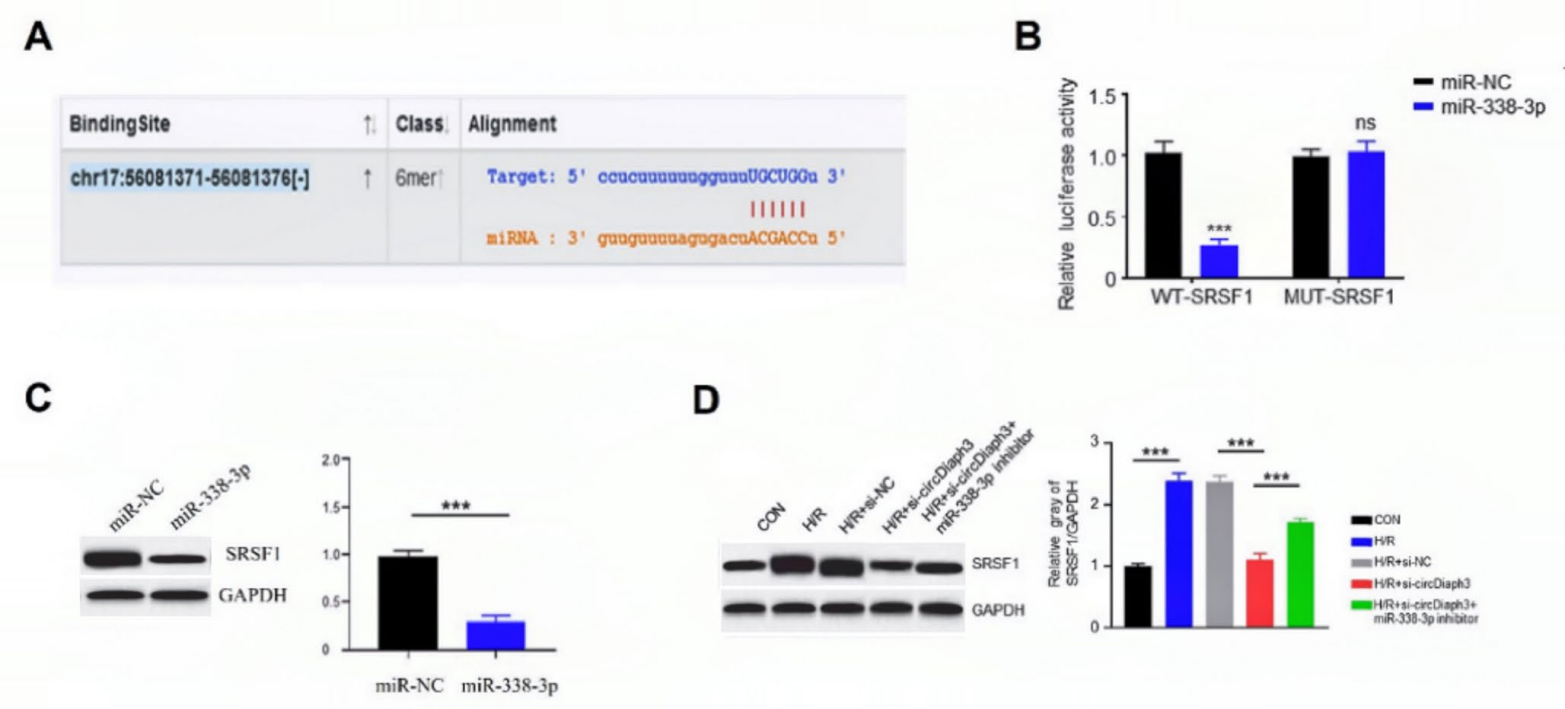
miR-338-3p targeted SRSF1 to inhibit its expression in H9C2 cells. **(A)** Wildtype and mutated binding sites for miR-338-3p in the 3’UTR of SRSF1. **(B)** Luciferase activity was used to examine the relationship between miR-338-3p and SRSF1. **(C)** WB was used to detect the protein level of SRSF1 in different groups of H9C2 cells (miR-NC, miR-338-3p). **(D)** The protein levels of SRSF1 in different groups of H9C2 cells (con, H/R, H/R + Si-NC, H/R + si-circDiaph3, H/R + si-circDiaph3 + miR-338-3p inhibitor) were detected by WB. *P < 0.05; **P < 0.01; and ***P < 0.001

To further explore potential downstream targets of miR-338-3p, a potential binding site for miR-338-3p in the 3’UTR of SRSF1 was predicted through bioinformatics (Fig. 5A). For luciferase assay, wild-type and mutant SRSF1 reporters were designed. In cells transfected with the wild-type SRSF1 reporter, overexpression of miR-338-3p decreased the luciferase activity, but not in cells transfected with the mutated SRSF1 reporter (Fig. 5B). Additionally, miR-338-3p mimics reduced SRSF1 expression in H9C2 cells (0.25 ± 0.07) (Fig. 5C). Also, after H/R treatment in H9C2 cells, SRSF1 expression was markedly increased when compared to control cells, but circDiaph3 silencing partially reduced SRSF1 expression (1.04 ± 0.04). Interestingly, concomitant silencing of miR-338-3p reversed this effect (Fig. 5D). These findings suggest that SRSF1 is a target of miR-338-3p.

### Overexpression of SRSF1 partially reverses the effect of mir-338-3p in H/R-induced H9C2 cells

**Fig. 6:**
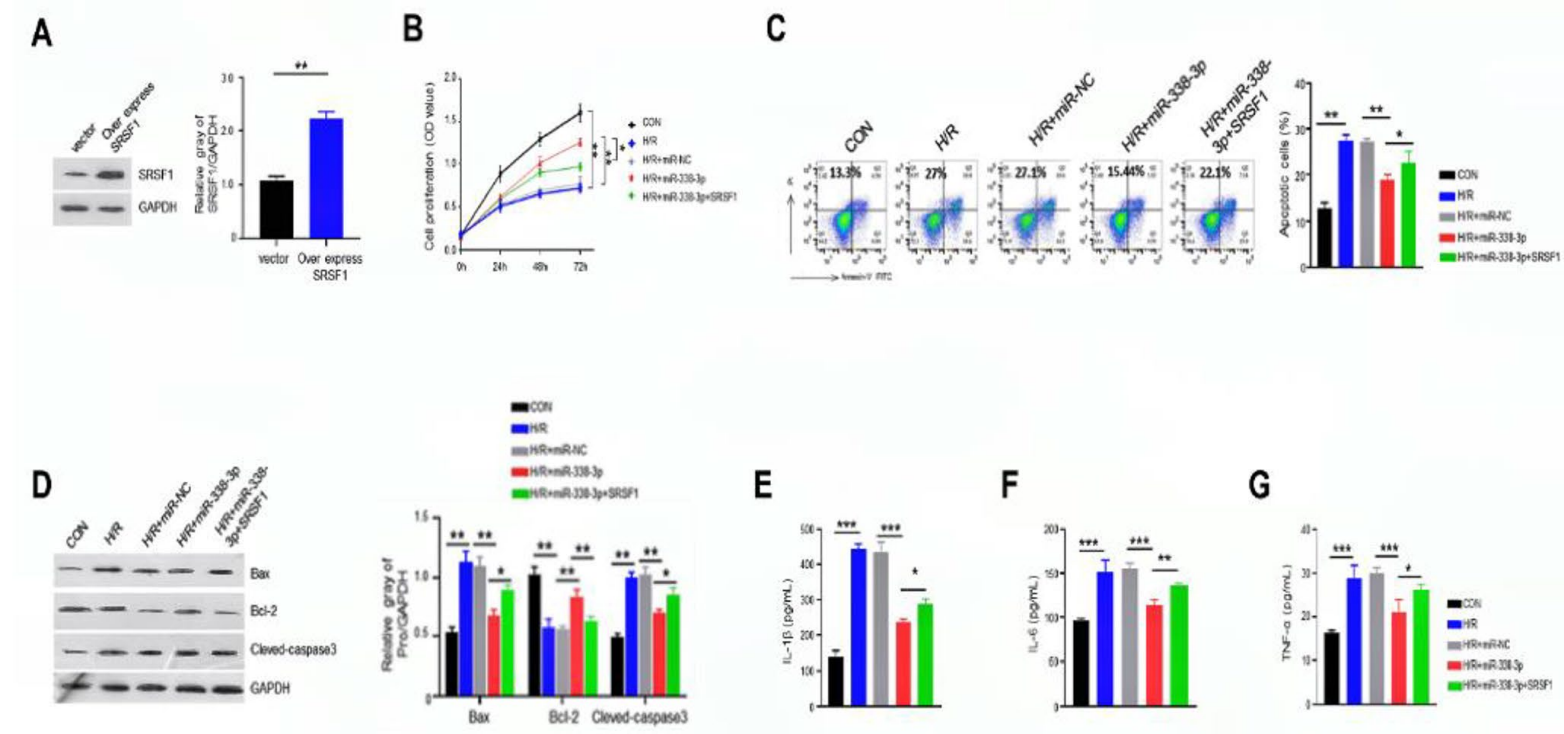
Overexpression of SRSF1 partially reversed the effect of miR-338-3p in H/R-induced H9C2 cells. **(A)** Western blot was used to detect the protein level of SRSF1 in different groups of H9C2 cells (empty-vector and vector overexpressing SRSF1). **(B)** CCK-8 assay was used to detect the proliferation rate of H9C2 cells in different groups. **(C)** Annexin V-PI staining was used to detect apoptosis in different groups of H9C2 cells. **(D)** Western blot was used to detect the protein expression levels of Bax, Bcl-2, and cleaved-Caspase-3. Elisa was used to measure the concentration levels of **(E)** IL-1β; **(F)** IL-6; **(G)** TNF-α in different groups of H9C2 cells. *P < 0.05; **P < 0.01; and ***P < 0.001

Finally, we investigated whether overexpressing SRSF1 could counteract the effects of miR-338-3p overexpression on cell apoptosis and inflammatory response following H/R treatment. To this end, we overexpressed SRSF1 in circDiaph3-overexpressing H9C2 cells and found increased expression of SRSF1 (23.31 ± 2.22) (Fig. 6A). We then used CCK-8 method to measure the proliferation of H9C2 cells in different groups (con, H/R, H/R + miR-NC, H/R + miR-338-3p, H/R + miR-338-3p + SRSF1). Result demonstrated that there was less proliferation of H9C2 cells after H/R induction, which was significantly increased after overexpressing miR-338-3p in these cells. Notably, when we co-transfected H9C2 cells to overexpress both miR-338-3p and SRSF1, it prominently reduced the proliferation capacity of miR-338-3p overexpressing cells (Fig. 6B). Additionally, overexpressing SRSF1 promoted the apoptosis in H9C2 cells that was inhibited by miR-338-3p overexpression in H/R-induced H2C9 cells (Fig. 6C). In H/R-treated H9C2 cells with miR-338-3p overexpression, Bcl-2 was upregulated (0.90 ± 0.11) while Bax and cleaved-caspase 3 were downregulated (0.68 ± 0.06, and 0.72 ± 0.05, respectively). Interestingly, concomitant SRSF1 overexpression reduced Bcl-2 expression while increased the expression of cleaved-caspase 3 and Bax (Fig. 6D). Furthermore, overexpression of miR-338-3p suppressed H/R-induced IL-1β, IL-6, and TNF-α secretion, whereas overexpression of SRSF1 abrogated these effects (284.36 ± 12.48, 139.62 ± 14.26, and 27.73 ± 8.39, respectively) (Fig. 6E-G). These results suggest that miR-338-3p mediates its function via regulating SRSF1.

## Discussion

Myocardial injury is an important pathological basis for many cardiovascular diseases, including coronary heart disease and myocardial infarction, among others (Chapman et al. 2017; Thygesen et al. 2018). Studying the molecular mechanism of myocardial injury will certainly provide effective targets and theoretical support for the treatment of myocardial infarction. Several studies have shown that circRNAs are actively involved in the pathogenesis and progression of myocardial infarction (Zhao et al. 2021). In this context, some research progress on the role of circRNAs in cardiovascular disease indicated that circRNAs could be used as therapeutic targets and clinical markers (Zhou et al. 2022). In one study, 158 circRNAs in human heart tissue were screened, and 12 of them were reported to be potential biomarkers of cardiac origin (Schulte et al. 2019).

Recently, circDiaph3 has been found to play an important role in rat vascular smooth muscle cell (VSMC) proliferation, differentiation, and migration (Xu et al. 2019). In this study, the authors found that circDiaph3 promotes VSMC differentiation towards contractile type promoting intimal hyperplasia. However, the role of circDiaph3 in H/R-induced myocardial injury is still not known, therefore, we conducted the present study. Our results demonstrated that H/R treatment could significantly increase the expression of circDiaph3 in heart tissue. Interestingly, circDiaph3 silencing inhibits H/R-induced apoptosis and inflammation in cardiomyocytes and reduces ROS expression levels, which is consistent with the results of previous publications (Liao et al. 2023; Ma et al. 2023).

MiRNAs, as downstream targets of circRNAs, have been shown in numerous studies to play important roles in H/R injury. miR-338-3p is a kind of miRNA specifically expressed in the heart, which has been shown as a therapeutic target in cardiac fibrosis (Huang et al. 2022). Existing studies have shown that miR-338-3p is involved in heart failure, myocardial fibrosis, myocardial ischemia, myocardial hypertrophy, and other heart diseases. For example, miR-338 has been shown to inhibit cardiomyocyte apoptosis and improve cardiac function in rats with myocardial infarction (Fu et al. 2020). Another study confirmed that miR-338-3p/RPS23 was involved in the progression of coronary heart disease (Qi et al. 2022). Several other studies have demonstrated the involvement of miR-338-3p in various diseases including cardiac pathologies (Wei et al. 2020; Sun et al. 2018; Yu et al. 2020; Chen et al. 2021). In line with these publications, our results also indicated that H/R could reduce the expression of miR-338-3p in myocardial infarction cells and enhancing the expression of miR-338-3p could reduce the expression of apoptotic proteins and inflammatory factors induced by H/R. Furthermore, we found for the first time that miR-338-3p was the down-stream target of circDiaph3 that mediated its function via sponging miR-338-3p to regulate H/R injury. All these miR-338-3p mediated functions are regulated by circDiaph3 suggesting that circDiaph3 may be involved in regulating biological processes such as cardiomyocyte apoptosis, inflammatory response, and oxidative stress. Therefore, the increased expression of circDiaph3 in acute myocardial infarction and hypoxic reperfusion may be an adaptive response that helps cardiomyocytes to cope injury and restore its function.

Several downstream targets of miR-338-3p have been identified, including MACC1, Met, MAP3K2, and SIRT6 (Zhang et al. 2019, 2022a, b; Jiang et al. 2021). SRSF1 has been shown to play an important role in mRNA metabolism, genomic stability, cell viability, and cell cycle progression (Das and Krainer 2014; Li et al. 2005; Li and Manley 2005). Moreover, several studies have revealed that SRSF1 is upregulated in different types of human cancer, and when overexpressed, it has the ability to drive the oncogenic transformation of fibroblasts and epithelial cells through increased proliferation and decreased apoptosis (Anczuków et al. 2012; Karni et al. 2007). In this report, SRSF1 was identified as a novel target of circDiaph3/miR-338-3p. We found that SRSF1 expression was high in H/R-induced injury and was the downstream target gene of miR-338-3p. CircDiaph3 mediated H/R-induced inflammation and apoptosis by sponging miR-338-3p via SRSF1. The reasons for the increased expression of circDiaph3 in AMI might be due to the fact that AMI patients have myocardial ischemia and hypoxia due to coronary artery stenosis or obstruction that resulted in increased circDiaph3 levels in the blood. At the same time, it has been known that AMI triggers an inflammatory response, thus many inflammatory factors might have induced upregulation of the circDiaph3 expression. In addition, it might be associated with myocardial tissue remodeling and neovascularization.

Nevertheless, further research is needed to investigate the downstream signaling pathway of SRSF1 in regulating myocardial H/R injury. Moreover, only miR-338-3p and SRSF1 were studied in this report. However, it is unclear whether other miRNAs and genes are involved in circdiaph3-mediated H/R-induced cardiac injury as circRNA can have many targeted miRNAs and miRNAs can also have many target genes. Future research will focus on identifying additional miRNAs and genes that may function as circDiaph3 downstream targets.

## Conclusions

In this study, we demonstrated for the first time that circDiaph3 aggravates cardiomyocyte apoptosis and inflammation in cardiac H/R injury through sponging miR-622 and enhancing SRSF1expression. Moreover, the current findings also suggest that miR-338-3pp/SRSF1 axis could be a potential therapeutic target for ameliorating H/R-induced myocardial injury. Notably, the present study was conducted only at the animal level, and we did not confirm the ability of the miR-338-3p/SRSF1 axis to exacerbate H/R-induced cardiomyocyte apoptosis and inflammation at a clinical level, so further clinical studies are needed to explore the mechanism by which the miR-338-3p/SRSF1 axis exacerbates H/R-induced cardiomyocyte apoptosis and inflammation.

## Data Availability

The datasets generated during and/or analyzed during the current study are available from the corresponding author upon reasonable request.
